# Supplementary material for: A LAT1-Like Amino Acid Transporter Regulates Neuronal Activity in the Drosophila Mushroom Bodies
Source: Cells. 2024 Aug 13;13(16):1340. doi: 10.3390/cells13161340 (PMC11352668; doi:10.3390/cells13161340)
Supplement: Supplementary file 1 [file cells-13-01340-s001.zip › cells-2931186-supplementary.pdf]

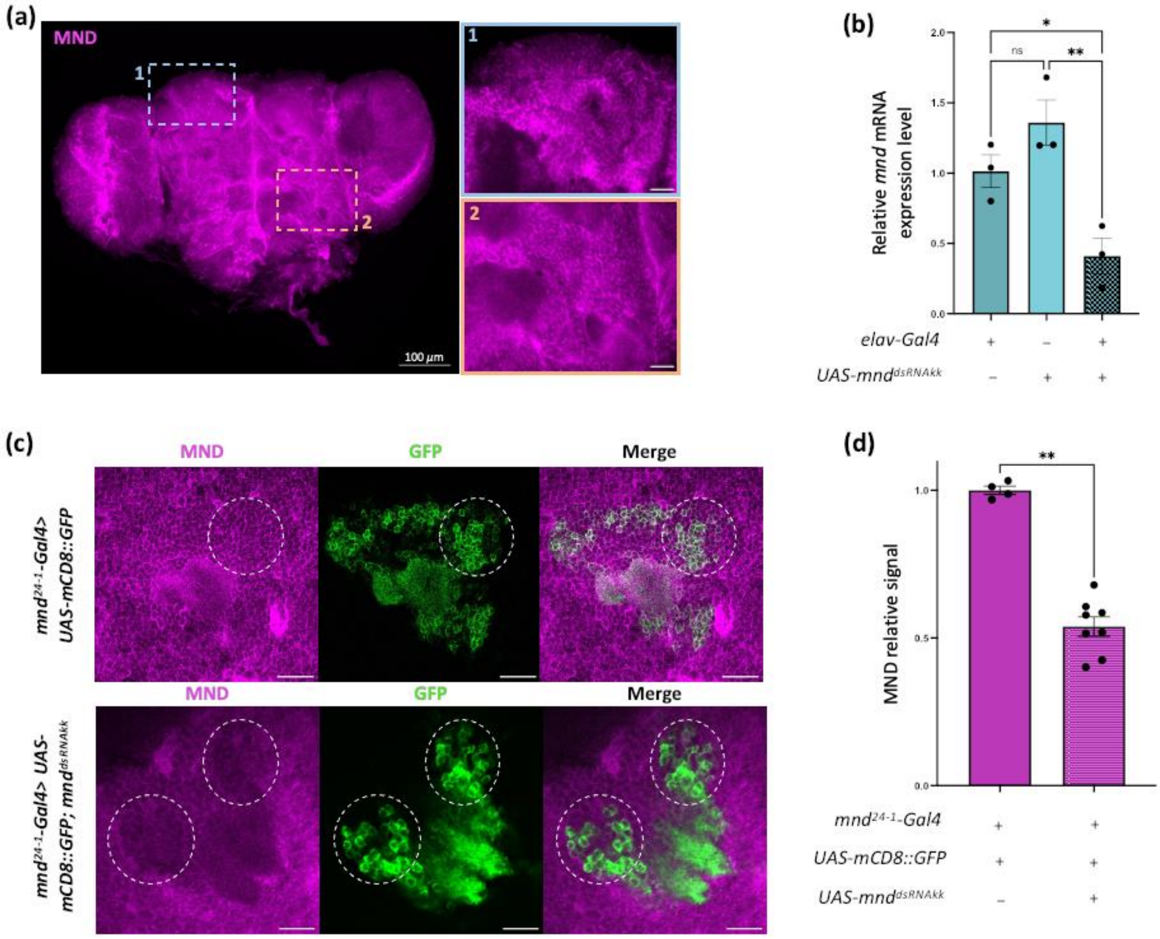

Figure S1. MND antibody specificity and *mnd* RNAi efficiency.

(**a**) Z-projection of whole brain (*mnd*$^{24-1}$*-Gal4>UAS-mCD8::GFP*) stained by anti-MND antibody. Scale bar, 100μm. Same brain confocal representative images of MND staining (magnification). 34 brains were examined. Scale bar, 20μm.

(**b**) Efficiency of the *mnd* RNAi. To confirm the efficiency of the *mnd* RNAi, *UAS-mnd*^{RNAiKK} was expressed in all neurons (*elav-Gal4>UAS-mnd*^{RNAiKK}*).* A 50% reduction in *mnd* mRNA was observed in the heads of flies (*elav-Gal4>UAS-mnd*^{RNAiKK}) compared with control flies (*elav-Gal4*>+ or *UAS-mnd*^{RNAiKK}>+). Each histogram represents the mean ± SEM of the relative *mnd* mRNA expression level determined by qRT-PCR. All individual data are shown by dots (n=3). Data were compared by ANOVA test. ns: not significant, ** : p<0.01 ; *** : p<0.001 ; **** : p<0.0001.

(**c**) Representative images of Kenon cells (green) from control brains (*mnd*$^{24-1}$*-Gal4>UAS-mCD8::GFP)* and *mnd* downregulated brains in MBs (*mnd*$^{24-1}$*-Gal4>UAS-mCD8::GFP;UAS-mnd*^{RNAiKK}) labeled by anti-MND (magenta). Scale bar, 20μm.

(**d**) Quantification of MND in *mnd*$^{24-1}$ positive Kenyon cells. Quantification of anti-MND immunoreactivity in control genotype brains (*mnd*$^{24-1}$*-Gal4>UAS-mCD8::GFP*) compared to *mnd* downregulated brains (*mnd*$^{24-1}$*-Gal4>UAS-mCD8::GFP;UAS-mnd*^{RNAiKK}). The signal was significantly reduced after RNAi-mediated knockdown specifically in Kenyon cells, confirming the specificity of the antibody. Each histogram represents the mean ± SEM of MND relative signal determine by the ratio of anti-MND immunoreactivity of Kenyon cells GFP positive. All individual data are shown by dots, n=4 and 8 brains. Statistics: ns: non-significant; *: p<0.05; **: p<0.01; ***: p<0.001; ****p<0.0001 (t test or Mann-Whitney test, depending on the Gaussian distribution of the data using D'Agostino & Pearson omnibus normality test).

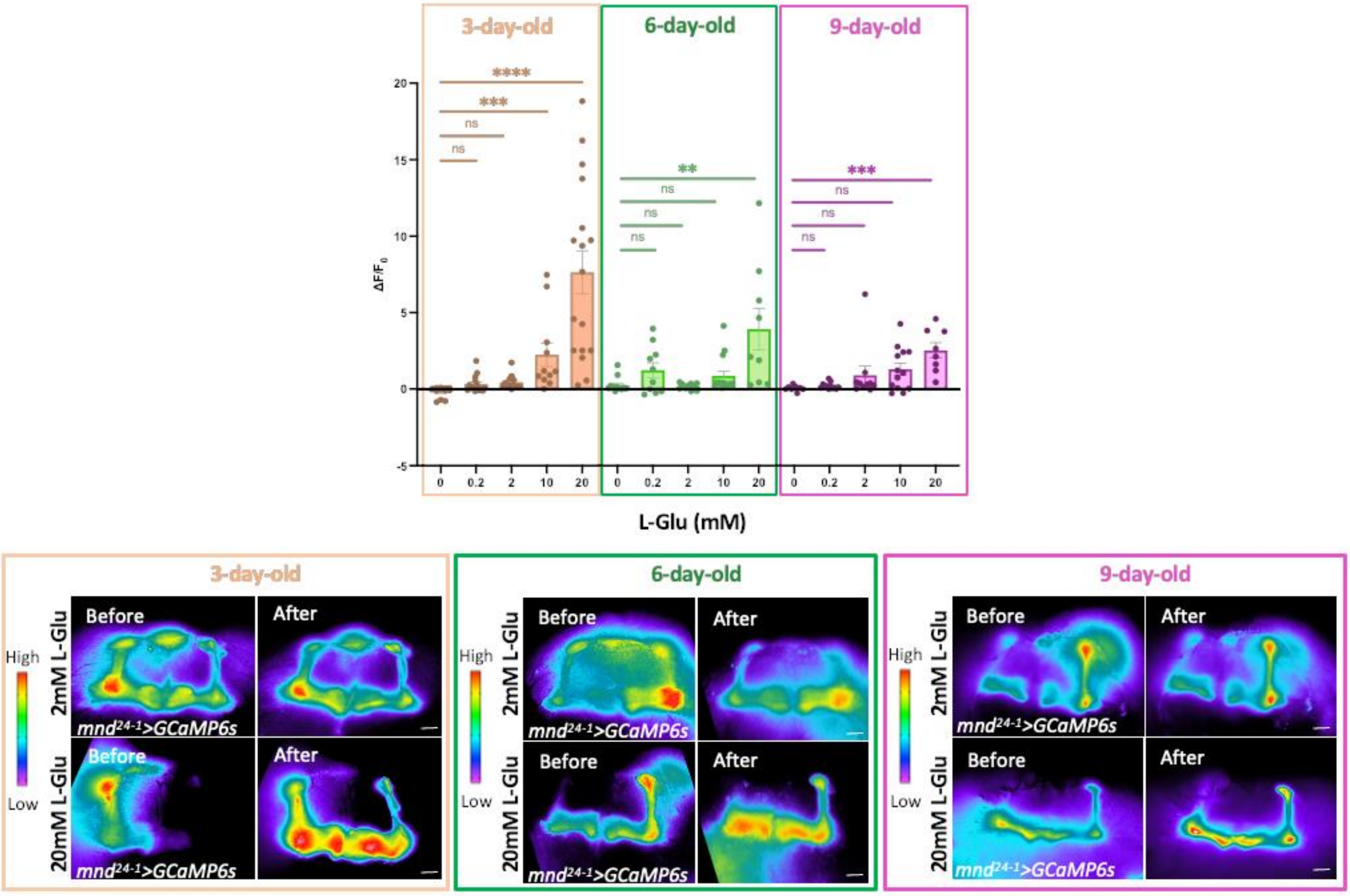

Figure S2. Dose and age-dependent MBs response to L-Glu.
Changes in neuronal activity of α/β and ɣ lobes in response to increased Glu concentrations (0 mM to 20 mM) in *mnd$^{24-1}$-Gal4>GCaMP6s* brains of 3-days to 9-days old flies. The maximum response of the MBs was observed in 3-day-old flies at a concentration of 20 mM. The response increases with the concentration of L-Glu at each age. Each histogram represents the mean ± SEM of the highest activity measured in the MBs. All individual data are shown by dots (n=8 to 17). For each age, all data were compared with the corresponding control (0 mM) by Kruskal-Wallis test. ns: not significant, ** : $p<0.01$; *** : $p<0.001$; **** : $p<0.0001$. Representative false-color images of *mnd$^{24-1}$-Gal4>GCaMP6s* brains showing fluorescence level before (basal activity) and after addition of L-Glu (2 or 20 mM) for three different ages (3-days, 6-days and 9-days old flies). The different intensities of basal GCaMP6s levels in the vicinity of the two lobes are sometimes difficult to convert to similar rainbow colors, and thus in some brains only one lobe appears in false color. Scale bar, 50μm.

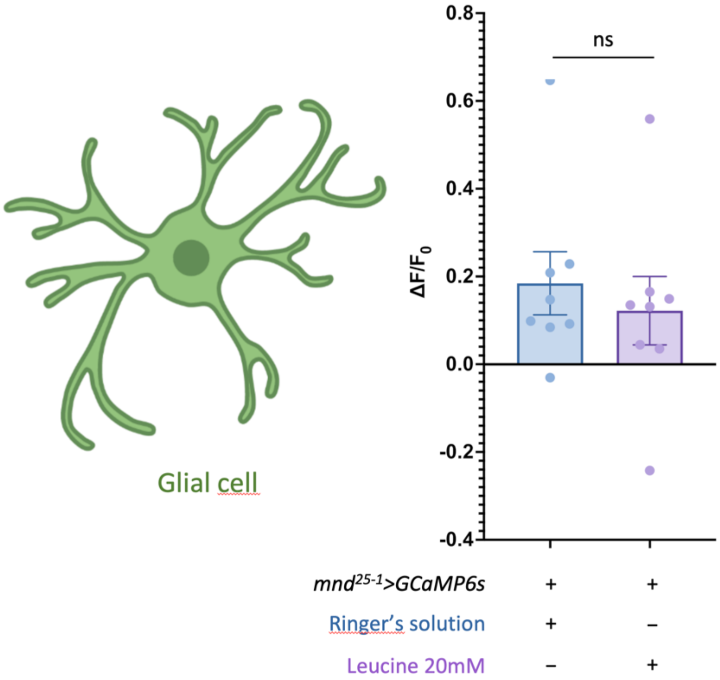

Figure S3. Leucine's lack of effect on glial calcium activity.
Real-time calcium imaging of *ex vivo* brains expressing a calcium sensor in glial cells in control brains (*mnd*$^{25\text{-}1}$*-Gal4>GCaMP6s)* of 3-day-old flies exposed to the Ringer's solution or 20mM of L-Leu. Each histogram represents the averaged fluorescence intensity of peaks ± SEM in glial cells. All individual data are shown by dots (n=8). Data were compared with the Ringer's solution control by Mann-Whitney test and were not significant.
